# Update on the Diagnosis and Management of Refractory Coeliac Disease

**DOI:** 10.1155/2013/518483

**Published:** 2013-05-09

**Authors:** Petula Nijeboer, Roy L. J. van Wanrooij, Greetje J. Tack, Chris J. J. Mulder, Gerd Bouma

**Affiliations:** Department of Gastroenterology, VU University Medical Center, De Boelelaan 1118, 1081 HZ Amsterdam, The Netherlands

## Abstract

A small subset of coeliac disease (CD) patients experiences persisting or recurring symptoms despite strict adherence to a gluten-free diet (GFD). When other causes of villous atrophy have been excluded, these patients are referred to as refractory celiac disease (RCD) patients. RCD can be divided in two types based on the absence (type I) or presence (type II) of an, usually clonal, intraepithelial lymphocyte population with aberrant phenotype. RCDI usually runs a benign course and may be difficult to be differentiated from uncomplicated, slow responding CD. In contrast, RCDII can be defined as low-grade intraepithelial lymphoma and frequently transforms into an aggressive enteropathy associated T-cell lymphoma with dismal prognosis. This paper describes the clinical characteristics of RCDI and RCDII, diagnostic approach, and the latest insights in treatment options.

## 1. Introduction 

Coeliac disease (CD) is a chronic enteropathy that occurs in genetically predisposed individuals in response to gluten ingestion and results in small intestinal villous atrophy that causes malabsorption in most cases [1]. Diagnosis relies on the demonstration of villous atrophy with an increased intraepithelial cell count in duodenal biopsies and is supported by the detection of serum IgA autoantibodies against transglutaminase (TGA) and endomysium (EMA). The only accepted treatment for CD is a life-long gluten-free diet (GFD), which interrupts the immune response triggered by gluten. Most patients report clinical improvement within weeks to months [1]. In a significant proportion of patients mucosal recovery lags behind and may last until 2 years after the instigation of a gluten-free diet [2–5]. The relevance of these findings is as yet unclear, but there are indications that these patients, despite symptom relief, suffer more often from osteoporosis and may be at increased risk to develop complicated forms of CD [5]. A small minority of patients does not show clinical improvement upon a GFD. The most common cause is inadvertent gluten contamination [3] or a (concomitant) small intestinal bowel disorder resembling CD. Patients are diagnosed with refractory celiac disease (RCD) when symptoms persist despite strict adherence to a GFD for over 12 months and other causes of villous atrophy have been excluded. This rare condition can occur in patients with persisting symptoms after initial diagnosis (primary resistance) or as recurring symptoms after initial response (secondary resistance), which can occur after years or even decades. RCD is divided in two types based on the absence (type I) or presence (type II) of an abnormal intraepithelial lymphocyte population referred to as aberrant lymphocytes [6]. These two groups are fundamentally different since RCD II, in contrast to RCD I, is considered as low-grade lymphoma that may evolve into aggressive enteropathy associated T-cell lymphoma in type II RCD with poor prognosis [7]. This paper describes the characteristics of RCDI and RCDII patients, diagnostic approach, and the latest insights in treatment options.

## 2. Epidemiology

RCD is mostly diagnosed around the age of 50 or thereafter but younger cases may be observed [2, 8]. Consistent with the predominance of CD in adult females, RCD occurs two to three times more often in women than in men [7, 9]. The exact incidence of RCDI and RCDII remains unknown, but both conditions appear to be rare. Different diagnostic criteria and differences in work up of RCD patients in referral centres make a valid comparison of these small subsets of patients difficult. One article reported that from a group of 713 CD patients only 5 patients (0.7%) were diagnosed with ulcerative jejunitis and thus presumably RCDII [10]. However, basing the diagnosis on aspects of ulcerative jejunitis might not reflect the true incidence of RCDII. A second study from a North American referral centre found an incidence of 1.5% for both RCDI and RCD II, the majority being RCD type I patients [11]. We have recently studied the prevalence of RCD I and II in the Netherlands and found 14 cases of RCDI and 20 of RCD II over a 6-year period, resulting in a cumulative incidence of 0.04 (unpublished data). 

## 3. Clinical Presentation

RCD patients may experience persisting symptoms (primary resistance) after diagnosis of CD and strict adherence to GFD for 12 months and this occurs almost exclusively in patients diagnosed with CD above the age of 50. In about 50% of patients, however, patients have developed recurring symptoms despite initial response to a GFD (secondary resistance) [12]. The most common symptoms in RCD include persistent diarrhoea, abdominal pain, and involuntary weight loss [13]. Moreover, fatigue, malaise, anaemia, hypoalbuminemia, vitamin deficiencies, and coexisting autoimmune disorders are frequently seen [13, 14]. The diagnosis RCDII becomes more likely when severe malnutrition, protein losing enteropathy, and ulcerative jejunitis are present [9].

Symptoms are notably less severe in RCDI, and endoscopic and histologic features are similar to those found in uncomplicated active CD. The diagnosis of RCDI may therefore be difficult and the distinction between a slow response to a GFD, inadvertent gluten ingestion, and RCD may be very difficult since there are no distinguishing criteria. In comparison with RCDII, malnutrition is usually less severe although there are exceptions. Ulcerative jejunitis is less frequently observed, and, when present, ulcerations are smaller and more limited in number when compared to RCDII [9]. 

## 4. Diagnostic Approach

### 4.1. Dietary Adherence and Initial CD Diagnosis

In the situation of nonresponsiveness to a GFD dietary adherence should be meticulously evaluated. Monitoring levels of TGA and/or EMA are generally suitable for this purpose; however, low levels of circulating autoantibodies do not necessarily exclude the diagnosis RCD since they may persist in the context of an ongoing autoinflammatory reaction that has become gluten independent [9]. In addition, all patients should be referred to a skilled dietitian with extensive experience in CD. When inadvertent gluten ingestion is reasonably excluded, the initial CD diagnosis should be re-evaluated. Absence of the CD-related genotypes (HLA-DQ2.5 or HLA-DQ8) [15] and/or absence of autoantibodies at time of initial CD diagnosis are highly suggestive of misdiagnosis [2]. 

### 4.2. Upper Gastrointestinal Endoscopy and Histological Evaluation

**Figure 1:**
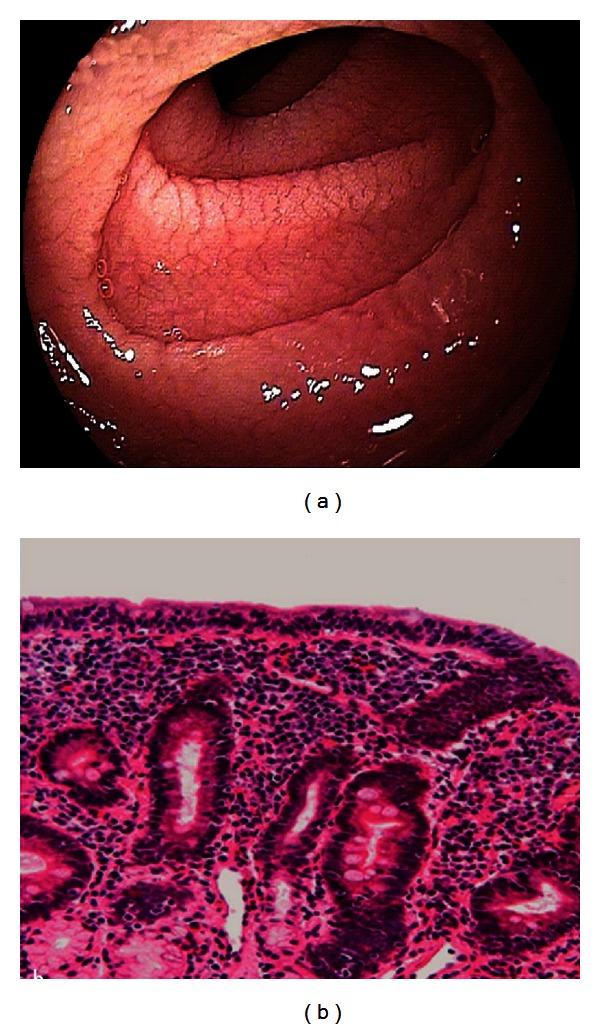
Endoscopic (a) and histologic (b) features found in RCD. Endoscopic abnormalities found in RCD include scalloped configuration of folds and fissuring with a mosaic pattern (a). Biopsies processed for histology show villous atrophy, crypt hyperplasia and increased infiltration of lymphoid cells in the epithelium (b).

Endoscopic assessment should include upper gastrointestinal endoscopy with extensive duodenal biopsies. Endoscopic features of RCD may be similar to those found in active uncomplicated CD [2] (see Figure 1). The finding of mucosal ulcerations in the jejunum is indicative of ulcerative jejunitis and supports the diagnosis RCD II [9, 16, 17].

When follow-up endoscopy reveals persisting villous atrophy, histological evaluation should focus on identifying other causes of villous atrophy such as Giardiasis, immunodeficiencies, collagenous sprue, Whipple's disease, and autoimmune enteropathy [18]. When these are excluded, the patient is diagnosed with RCD. Detailed analysis of biopsy samples using immunohistochemistry, flow cytometric analysis, and analysis of T-cell receptor rearrangement is mandatory to further categorise these patients.

### 4.3. Identification of Aberrant Intraepithelial Lymphocytes

Under physiological circumstances, the small intestine contains intraepithelial lymphocytes (IELs) interspersed between the epithelial cells. These cells markedly increase in number in uncomplicated CD as well as in RCD type I. They have a normal T-cell phenotype, characterized by the cell surface expression of CD3^+^CD8^+^ with a polyclonal T-cell repertoire. The majority of these cells carry the *αβ* T-cell receptor, although up to 15% of IELs carry the *γδ* T-cell receptor, a number that may increase to 40% in active CD [19]. Under physiological circumstances a small proportion of IELs consists of cells lacking surface CD3 and generally CD8 but expressing intracellular CD3 (iCD3). Such cells typically constitute <10% of IELs, but in RCD II a massive expansion of these cells is found, in some cases to be more than 90% of the IEL compartment [20]. A cutoff of >20% of aberrant cells is indicative for RCD II and may be used to separate this disease entity from RCD I and other forms of villous atrophy [21]. This is of relevance since RCD II can be considered as a low-grade lymphoma that is able to evolve to an aggressive enteropathy-associated T-cell lymphoma (EATL) (see later). The latter carries a dismal prognosis, and early identification of the premalignant aberrant cells provides a window of opportunity to prevent these cells to evolve into an overt lymphoma.

Such aberrant cells can be identified by conventional immunohistochemical analysis and by flow cytometry. Immunohistochemical analysis is an easy applicable technique but is unable to distinguish surface expression from iCD3. The distinguishing feature relates to the fact that in uncomplicated CD and RCDI the majority of CD3^+^ IELs are also CD8 positive, while in RCDII patients most of iCD3^+^ IELs are CD8 negative [20]. Thus by subtracting the CD8^−^CD3^+^ cells from the total amount of CD3-positive cells, the number of aberrant cells can be estimated. This easy applicable technique, however, lacks sensitivity and specificity [22]. A major pitfall relates to the high number of CD8^−^ gamma-delta (*γδ*) T cells in active celiac disease giving rise to false-positive results. 

**Figure 2:**
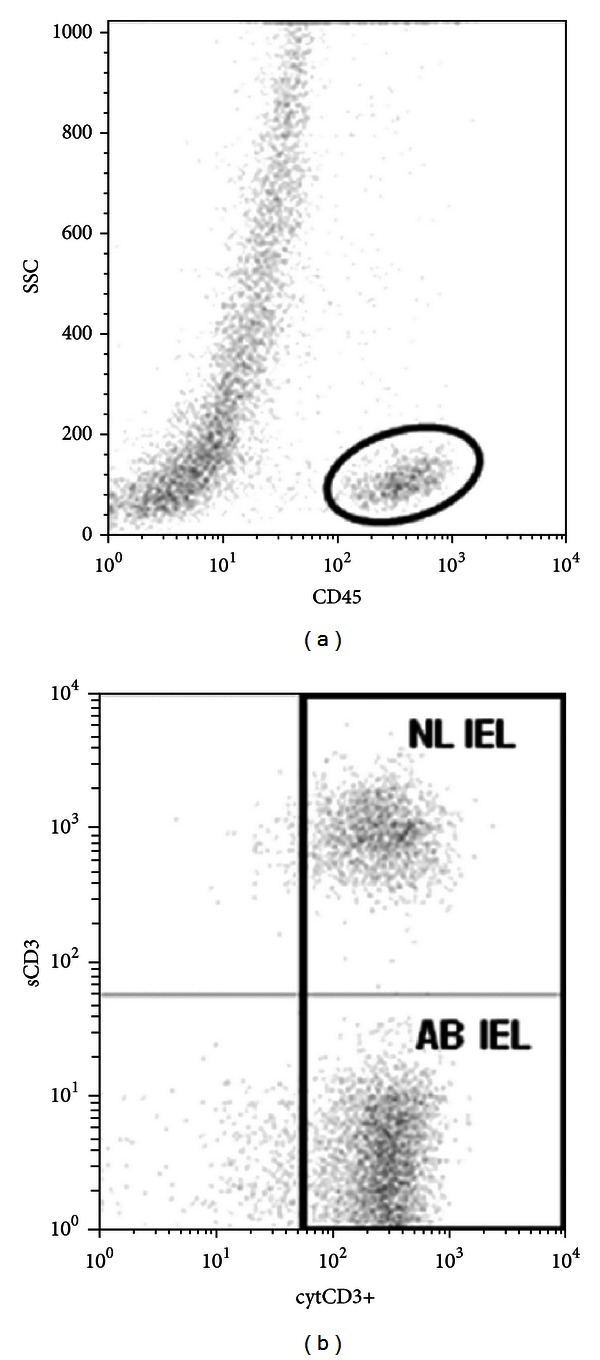
Flow cytometric analysis to identify aberrant and normal IELs. Cells with a strong CD45 expression and low to intermediate forward and sideward scatter were selected (a), after which IELs expressing intracellular CD3 expression were used for further studies (b). Ab IEL: aberrant IEL population (sCD3-cytCD3+): NL IEL: normal IEL population (sCD3 + cytCD3+).

Flow cytometric analysis, contrary to immunohistochemistry, is able to differentiate cytoplasmatic from membranous CD3 expression (see Figure 2). In addition, this technique is able to identify other relevant cell populations such as *γδ* T cells or other “aberrant” IEL with preserved CD8 expression. This technique has been clinically validated and shown to be superior to T-cell receptor (TCR) clonality analysis in identifying patients at risk to develop an EATL [22]. 

It has been postulated that RCD type II constitutes a low-grade lymphoma and that the expansion of aberrant cells occurs as the consequence of clonal expansion of such cells. Consequently identification of a monoclonal pattern upon TCR rearrangements analysis may contribute to the diagnosis of RCD II, and indeed it has been suggested that to ascertain the diagnosis of RCDII requires the demonstration of a clonal rearrangement of the TCR [19]. In the study by Malamut et al. it was found that 97% of patients characterized as RCD II based on the presence of increased numbers of aberrant cells above 50% by immunohistochemistry displayed clonality of the TCR*γ* chain versus 0% of RCD I patients [9]. In our experience, however, clonality analysis lacks sensitivity and specificity and is of limited value to separate RCD type I from type II [23].

### 4.4. Imaging Techniques

**Figure 3:**
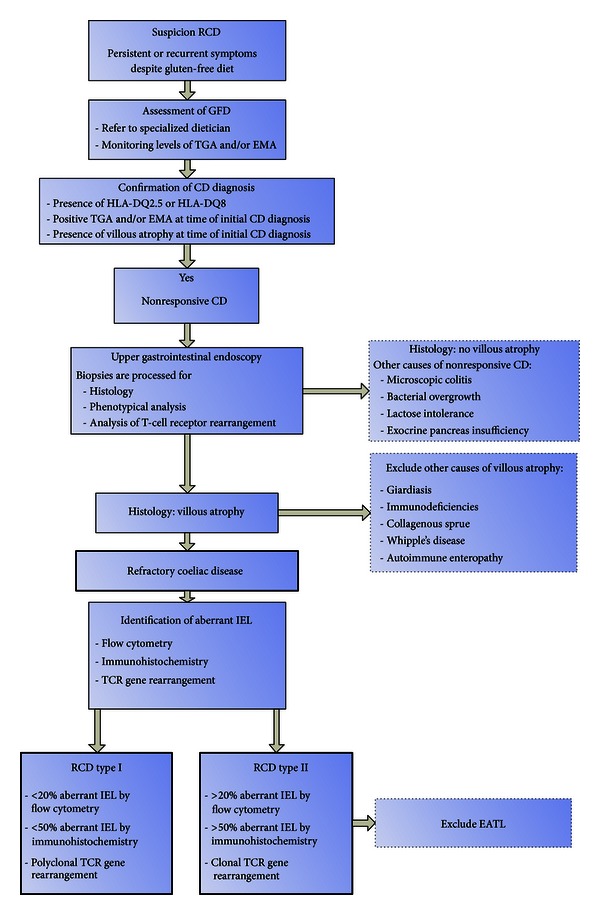
Algorithm for diagnostic approach of RCD.

Abdominal CT scan can be useful in the diagnostic process of RCD. Mesenteric lymphadenopathy, bowel-wall thickening, and spleen atrophy are more commonly detected in patients with RCDII and EATL compared to RCDI or uncomplicated CD [24]. Other diagnostic tools which might be helpful in the diagnostic work-up of RCD patients include videocapsule enteroscopy (VCE), MR enteroclysis, and double balloon enteroscopy (DBE) which all allow visualization of intestinal lesions. VCE is useful in determining the extent of lesions and is less invasive than other endoscopic techniques [16, 25]. Comparison of VCE with MR enteroclysis indicates that both modalities are complementary in diagnostic accuracy in the analysis of small-bowel disease [26]. However, VCE appears of low diagnostic yield in RCDI [27]. In addition, it should be kept in mind that VCE has an inherent risk of retention, in particular in ulcerative jejunitis where stenoses are common. DBE can efficiently detect or exclude suspected lesions beyond the reach of the standard endoscopy, especially when suggested by other imaging modalities such as abdominal CT scan [17]. For identifying suspicious lesions for EATL or assessment of probable ulcerative jejunitis DBE is superior as compared to other imaging techniques [14]. Finally, PET scan is useful to eliminate an invasive lymphoma. See Figure 3 for an algorithm for the diagnostic approach of RCD.

## 5. Pathogenesis

### 5.1. Predisposing Factors

The question as to why a small minority of CD patients develops RCD remains to be determined. In addition, it is unknown whether this can be pinpointed to any of the known genetic risk factors for CD. Among the 39 genetic risk factors that have been identified in CD, the major histocompatibility complex (MHC) alleles harbour the strongest genetic association [28]. Approximately 95% of CD patients express genes encoding the MHC class II protein HLA-DQ2.5 versus 30% of the control population, and the majority of the remaining patients are HLA-DQ8 positive [29]. By presenting gluten peptides to immune cells, the HLA molecules are key players in driving the gluten-specific immune response in CD. The observation that RCD type II and EATL show a strong association with HLA-DQ2 homozygosity suggests that the strength of the gluten-specific T-cell response in the intestinal epithelium influences RCDII and EATL development [30]. Consequently, this indirectly suggests that adherence to a GFD, especially in DQ2.5 homozygous or DQ2.5/DQ8 compound heterozygotes patients, might affect the risk of developing RCD and/or EATL [31]. However, it should be noted that this is speculative and in a recent Swedish study the association between poor compliance and an increased risk of EATL could not be found [32]. 

So far it is unknown whether any of the other identified genetic risk factors for CD is involved in the susceptibility to RCD or EATL but preliminary findings from a genome-wide study in European CD patients found that none of the known celiac disease susceptibility variants showed association with RCDII, suggesting that the RCDII phenotype is due to different genetic factors. (van Wanrooij, personal communication with Prof. C. Wijmenga, Groningen, the Netherlands).

### 5.2. Pathogenesis of RCDI

The pathogenesis of RCDI is enigmatic; however, by definition, the intestinal immune reaction initially induced by gluten has evolved into an autonomous (auto)immune reaction. This also explains why most RCDI patients improve under immunosuppressive treatments. It should be kept in mind that the distinction between slow responding CD and RCD type I may be difficult especially in the case of low persisting levels of circulating autoantibodies. In our experience a substantial number of (especially older) patients initially suspected for RCD improved spontaneously after longer follow-up indicating slow response rather than refractoriness [33]. In addition, there are data to suggest that RCD I may compromise a heterogeneous group of patients. This is exemplified by the observation that in a subset of RCD I patients a thickened subepithelial collagen layer is found which is indicative for collagenous sprue (CS) [19]. Whether or not this defines a subgroup of RCD I patients with different immunopathogenesis remains to be determined. 

There are currently limited data available on the immune mechanisms involved in the development of RCD I. In an attempt to shed light on this issue, we studied cell subsets in the epithelium of RCDI patients and cytokine profile in the peripheral circulation and found these to be similar to uncomplicated active CD but dissimilar from RCDII patients [34, 35].

It was recently postulated that IL-15, through impairment of TGF-*β* signaling and inhibition of FoxP3^+^ CD4^+^CD25^+^ regulatory T-cell activity, impairs control of autoreactive cells that consequently accumulate and ultimately sustain an intestinal immune response that becomes independent of gluten intake [19]. Unpublished data indeed suggest that some but not all RCDI patients have markedly increased serum levels of IL-15.

### 5.3. Pathogenesis of RCDII

The hallmark of RCDII is the expansion and accumulation of IEL with an aberrant phenotype [20]. These cells have been characterized recently in great detail and were found to be lineage negative cells (i.e., lacking the cell surface markers CD3, CD14, CD19, and CD56) in combination with intracellular CD3 that is distinct from T, B, NK, and lymphoid tissue inducer cells. They can constitute up to 10% of the IEL compartment of patients without CD, and in higher frequencies in children, and are also found in the thymus. They may represent the physiological counterpart of aberrant cells expanded in RCDII and transformed in RCDII-associated lymphoma [36]. Further immunophenotyping revealed that aberrant IELs display different stages of maturity between RCDII patients, of which only the patients harbouring the most mature aberrant IEL population developed an EATL [22]. Of relevance for understanding their role in RCD and lymphomagenesis is the observation that these cells express a functional interleukin-15 (IL-15) receptor. This fits in a model where massive overproduction of IL-15 by enterocytes leads to continuous activation of IELs. Indeed there is evidence that this cytokine is upregulated in patients with RCD II [37]. The source of this increased IL-15 response is as yet unknown but might be related to IFN-*α*, which can induce the production of IL-15 during chronic viral infection [38]. There is indeed some evidence to suggest that chronic viral infections are found in a substantial number of patients [19]. The increased IL-15 response results in the expression of cytotoxic proteins and stimulates production of IFN-*γ* and NKG2D-dependent cytotoxicity against enterocyte lines. MICA is one of the NKG2D ligands and is strongly upregulated at the epithelial surface of enterocytes in RCDII patients. In this model, RCDII IELs activated by enterocyte-derived IL-15 exert cytotoxicity against epithelial cells and are responsible for the severe enteropathy observed in RCDII patients. The strong anti-apoptotic effect of IL-15 finally might explain the accumulation and eventually expansion of these cells despite their low in situ proliferative capacity [19]. The concept of normal cells losing apoptotic control as the consequence of increased antiapoptotic signals is tempting and suggests a multistep model where aberrant cells survive due to increased production of antiapoptotic cytokines. In a next step towards lymphomagenesis a subset of cells undergo clonal expansion and finally when chromosomal aberrations have occurred these cells transform towards a lymphoma.

## 6. Treatment

RCDI and RCDII are rare diseases, and to date, there is no standardised therapy. The choice of treatment strategy is mostly guided by observational studies and only small cohorts have been described. Particularly in RCDII, severe wasting and protein losing enteropathy are frequently seen and total parenteral nutrition and substitution of vitamins may be necessary.

### 6.1. Treatment of RCDI

Management of a RCDI patient relies on a combination of nutritional support and immunosuppressive treatment. Immunosuppressive drugs suggested for RCDI include steroids, thiopurines, and infliximab. Steroids, either in the form of topical budesonide or as systemic steroids, suppress clinical symptoms in RCDI, and clinical improvement is reported in up to 90% of patients [8, 9, 39, 40]. However, histological response can only be seen in a small subset of patients, and moreover, corticosteroid dependence is usual in RCDI [8, 39, 40]. Combination therapy of azathioprine and prednisone might exert better histological restoration although complete normalization of villi is only seen in 50% of patients [8, 41]. Treatment with infliximab may induce clinical and histological response, but so far only case reports have been described [42, 43]. In our centre, tioguanine has been successfully applied. It has a small spectrum of side effects and it has good intestinal absorption despite villous atrophy. Our recent published data showed that 10 patients tolerated long-term tioguanine. Of those, clinical and histological response was observed in 83% and 78%, respectively [44]. Although there is some concern that thiopurines might enhance the risk for development of lymphoma, we have not observed progression to lymphoma in a group of 43 RCD I patients with a mean follow-up of 72 months [7]. Finally, the observation that this drug maybe associated with the risk of nodular regenerative hyperplasia of the liver should not be ignored.

### 6.2. Treatment of RCDII

The treatment of RCDII remains a challenge. As in RCDI, nutritional deficiencies and metabolic disorders should be corrected. In contrast to RCDI, there is no place for immunosuppressive drugs in the treatment of RCDII. Although corticosteroids might exert clinical effects, this has no influence on the onset of EATL and especially does not exclude underlying EATL [12]. In a descriptive study of Malamut et al. of 16 RCDII patients who developed EATL, 10 had received immunosuppressants [9]. Moreover, azathioprine might enhance the risk or accelerate the onset of EATL [9, 41, 45]. Combination therapy of azathioprine and prednisone in RCDII patients showed development of EATL in 7 of the 8 treated patients [41]. 

Given the high percentage of RCD II patients that develop an EATL, the treatment goal in RCD is to destroy the aberrant cell population before they transform to a lymphoma. Aberrant IELs are cells with a low proliferative capacity, and therefore antiproliferative drugs have no proven value in this disease. Cladribine (2-chlorodeoxyadenosine (2-CDA)) is a synthetic purine nucleoside homologue being equally toxic to proliferating as to nondividing lymphoid cells [46]. Because of this unique feature it is supposed to be especially active against low-grade malignancies, including hairy cell leukaemia and for similar reasons may be effective in RCD type II. 

In a series of 32 patients, treatment with 2-CDA was well tolerated and 14 displayed clinical and histological remission, and another 4 displayed clinical improvement [47]. This was accompanied by a reduction of aberrant cells in 40% of patients. The 3- and 5-year survival rates were 83% in the responding group and 63% and 22% in the nonresponding group, respectively. However, 2-CDA has no curative effect on EATL, and therefore adequate exclusion of EATL should be performed before treatment is started [9, 45, 48]. These encouraging data should however be seen in the light of the explosive onset of overt lymphoma a few weeks after 2-CDA treatment observed in two patients [9]. 

One possible alternative treatment strategy includes autologous hematopoietic stem cell transplantation (auto-SCT). High-dose chemo/radiotherapy followed by auto-SCT has been an effective therapy for refractory disease not only in hematological malignancies, but also in severe autoimmune disease [49–52]. In a series of 18 patients not responding to 2-CDA, 13 underwent auto-SCT with a 4-year survival rate of 66%. Quite surprisingly, no significant sustained reduction of abnormal IEL in the treated patients could yet be shown, and therefore long-term outcome of this treatment, notably the onset of EATL, is warranted (Table 1) [53].

## 7. Complications/Follow-Up

RCDI generally runs a benign course and this is also reflected in five-year survival rates ranging between 80–96% [7, 8]. Main causes of death in this group were nutrition related, and, in one study, lymphoma was observed in an occasional patient [9]. It should be noted that lymphoma development in this category of patients was not observed in two other studies [7, 8, 14]. 

RCDII on the other hand is associated with a poor prognosis with a 5-year survival between 44 and 58% [7–9]. The higher mortality associated with RCDII can be largely attributed to the much higher risk of developing EATL which occurs between 33% to 52% within 5 years after diagnosis [7]. The outcome of EATL remains poor with a 5-year survival of only 8–20% [55, 56]. Regular follow-up, including upper gastrointestinal endoscopy, CT scan or MR enteroclysis, and PET scan, is necessary to screen RCDII patients and to detect EATL as early as possible.

## 8. Conclusion and Future Perspectives

During the last decade, significant progress has been made in understanding the biological basis of refractory celiac disease. RCD can be divided in two types based on the absence (type I) or presence (type II) of an abnormal intraepithelial lymphocyte population that is generally clonal in nature. This cell population is found under physiological circumstances in the intestine and may expand as the consequence of a lack of apoptotic control. Clonal expansion of these cells is indicative for a low-grade intraepithelial T-cell lymphoma that may evolve into an aggressive overt EATL. RCD type I and II differ substantially in clinical presentation, histology, endoscopic characteristic with a generally benign course, and good prognosis in the former and a poor prognosis in the latter which can be attributed to a high risk to develop EATL. Currently there are no standardized treatment regimens, but the identification of the antiapoptotic pathway mediated by IL-15 may provide novel treatment avenues for this devastating disorder.

## Figures and Tables

**Table 1:** Summary of treatment modalities evaluated in RCD.

Therapy	No. of patients	Therapeutic effect	Notes and side effects	Reference
Thioguanine	12 RCDI	10 patients tolerated TG. clinical and histological response was observed in 83% and 78% respectively	1 patient died within 4 months of therapy due to progression of RCDI. Side effects: muscle spasms, elevation of biochemical liver tests	[44]

Azathioprine and Prednisone	10 RCDI 8 RCDII	Clinical improvement in all patients in both groups. 8 RCD type I patients responded histologically, complete histological normalization in 4 patients	7 RCDII patients died from EATL	[41]

Mesalamine and Budesonide	10 RCDI	5 patients had complete symptom relief. No conclusion on histological improvement	4 patients had concomitant microscopic colitis. Side effect: headaches	[40]

Budesonide	23 RCDI 5 RCDII	Overall, 76% of the patients had a clinical response to budesonide, considered as complete response in 55%. No histological improvement in any patient. RCDII patients had persistent clonal proliferation of IELs	1 patient with RCDII died of sepsis and malnutrition. 7 patients had concomitant microscopic colitis. There were no serious adverse events reported	[39]

Infliximab	1 RCDI	Excellent clinical results. Treatment was continued over the following 2 years with a return to near normal histology	No serious adverse events reported	[43]

Infliximab	1 RCDI	Complete clinical improvement. Marked histological improvement	No serious adverse events reported	[42]

Cyclosporin	13 (no differentiation)	Clinical response in 8 patients. Normalisation of histology in 5 patients	No serious adverse events reported	[54]

Cladribine	32 RCDII	Clinical response was observed in 81%, complete histological response in 47% and immunological response in 41%. 5 year survival in those who responded was 83% compared to 22% in those who did not	In total, 12 of 32 patients died of whom 42% died of EATL	[47]

Autologous stem cell transplantation	18 RCDII	13 patients were feasible for auto-SCT and transplanted successfully. Majority showed clinical improvement. 5 patients showed compete histological remission. 4-year survival rate was 66%	In 5 patients, auto-SCT could not be performed; they all died with a median survival of 5.5 months. 1 patient died because of transplant-related complications. EATL was observed in one transplanted patient, after 4 years of follow-up	[53]

## References

[1] Tack GJ, Verbeek WHM, Schreurs MWJ, Mulder CJJ (2010). The spectrum of celiac disease: epidemiology, clinical aspects and treatment. *Nature Reviews Gastroenterology and Hepatology*.

[2] Al-Toma A, Verbeek WHM, Mulder CJJ (2007). Update on the management of refractory coeliac disease. *Journal of Gastrointestinal and Liver Diseases*.

[3] Wahab PJ, Meijer JWR, Mulder CJJ (2002). Histologic follow-up of people with celiac disease on a gluten-free diet: slow and incomplete recovery. *American Journal of Clinical Pathology*.

[4] Lanzini A, Lanzarotto F, Villanacci V (2009). Complete recovery of intestinal mucosa occurs very rarely in adult coeliac patients despite adherence to gluten-free diet. *Alimentary Pharmacology & Therapeutics*.

[5] Kaukinen K, Peräaho M, Lindfors K (2007). Persistent small bowel mucosal villous atrophy without symptoms in coeliac disease. *Alimentary Pharmacology & Therapeutics*.

[6] Mudler CJJ (2001). When is a coeliac a coeliac? Report of a working group of the United European Gastroenterology Week in Amsterdam, 2001. *European Journal of Gastroenterology and Hepatology*.

[7] Al-Toma A, Verbeek WHM, Hadithi M, von Blomberg BME, Mulder CJJ (2007). Survival in refractory coeliac disease and enteropathy-associated T-cell lymphoma: retrospective evaluation of single-centre experience. *Gut*.

[8] Rubio-Tapia A, Kelly DG, Lahr BD, Dogan A, Wu TT, Murray JA (2009). Clinical staging and survival in refractory celiac disease: a single center experience. *Gastroenterology*.

[9] Malamut G, Afchain P, Verkarre V (2009). Presentation and long-term follow-up of refractory celiac disease: comparison of type I with type II. *Gastroenterology*.

[10] West J (2009). Celiac disease and its complications: a time traveller's perspective. *Gastroenterology*.

[11] Roshan B, Leffler DA, Jamma S (2011). The incidence and clinical spectrum of refractory celiac disease in a north american referral center. *American Journal of Gastroenterology*.

[12] Rubio-Tapia A, Murray JA (2010). Classification and management of refractory coeliac disease. *Gut*.

[13] Al-Toma A, Verbeek WHM, Mulder CJJ (2007). The management of complicated celiac disease. *Digestive Diseases*.

[14] Daum S, Cellier C, Mulder CJJ (2005). Refractory coeliac disease. *Best Practice and Research*.

[15] Hadithi M, von Blomberg BME, Crusius JBA (2007). Accuracy of serologic tests and HLA-DQ typing for diagnosing celiac disease. *Annals of Internal Medicine*.

[16] van Weyenberg SJ, Smits F, Jacobs MA, van Turenhout ST, Mulder CJ (2012). Video capsule endoscopy in patients with nonresponsive celiac disease. *Journal of Clinical Gastroenterology*.

[17] Hadithi M, Al-Toma A, Oudejans J, van Bodegraven AA, Mulder CJ, Jacobs M (2007). The value of double-balloon enteroscopy in patients with refractory celiac disease. *American Journal of Gastroenterology*.

[18] Mulder CJJ, Wahab PJ, Moshaver B, Meijer JWR (2000). Refractory coeliac disease: a window between coeliac disease and enteropathy associated T cell lymphoma. *Scandinavian Journal of Gastroenterology, Supplement*.

[19] Malamut G, Meresse B, Cellier C, Cerf-Bensussan N (2012). Refractory celiac disease: from bench to bedside. *Seminars in Immunopathology*.

[20] Cellier C, Patey N, Mauvieux L (1998). Abnormal intestinal intraepithelial lymphocytes in refractory sprue. *Gastroenterology*.

[21] Al-Toma A, Verbeek WHM, Hadithi M, von Blomberg BME, Mulder CJJ (2007). Survival in refractory coeliac disease and enteropathy-associated T-cell lymphoma: retrospective evaluation of single-centre experience. *Gut*.

[22] van Wanrooij RLJ, Schreurs MWJ, Bouma G (2010). Accurate classification of RCD requires flow cytometry. *Gut*.

[23] Tack GJ, van Wanrooij RL, Langerak AW (2012). Origin and immunophenotype of aberrant IEL in RCDII patients. *Molecular Immunology*.

[24] Mallant M, Hadithi M, Al-Toma AB (2007). Abdominal computed tomography in refractory coeliac disease and enteropathy associated T-cell lymphoma. *World Journal of Gastroenterology*.

[25] van Weyenberg SJB, Turenhout STV, Bouma G (2010). Double-balloon endoscopy as the primary method for small-bowel video capsule endoscope retrieval. *Gastrointestinal Endoscopy*.

[26] van Weyenberg SJ, Bouman K, Jacobs MA (2013). Comparison of MR enteroclysis with video capsule endoscopy in the investigation of small-intestinal disease. *Abdom Imaging*.

[27] Daum S, Wahnschaffe U, Glasenapp R (2007). Capsule endoscopy in refractory celiac disease. *Endoscopy*.

[28] Trynka G, Hunt KA, Bockett NA (2011). Dense genotyping identifies and localizes multiple common and rare variant association signals in celiac disease. *Nature Genetics*.

[29] Wolters VM, Wijmenga C (2008). Genetic background of celiac disease and its clinical implications. *American Journal of Gastroenterology*.

[30] Al-Toma A, Goerres MS, Meijer JWR, Peña AS, Crusius JBA, Mulder CJJ (2006). Human leukocyte antigen-DQ2 homozygosity and the development of refractory celiac disease and enteropathy-associated T-cell lymphoma. *Clinical Gastroenterology and Hepatology*.

[31] di Sabatino A, Biagi F, Gobbi PG, Corazza GR (2012). How I treat enteropathy-associated T-cell lymphoma. *Blood*.

[32] Olen O, Askling J, Ludvigsson JF, Hildebrand H, Ekbom A, Smedby KE (2011). Coeliac disease characteristics, compliance to a gluten free diet and risk of lymphoma by subtype. *Digestive and Liver Disease*.

[33] van Wanrooij

[34] Verbeek WHM, Goerres MS, von Blomberg BME (2008). Flow cytometric determination of aberrant intra-epithelial lymphocytes predicts T-cell lymphoma development more accurately than T-cell clonality analysis in Refractory Celiac disease. *Clinical Immunology*.

[35] Tack GJ, van Wanrooij RL, von Blomberg BM (2012). Serum parameters in the spectrum of coeliac disease: beyond standard antibody testing—a cohort study. *BMC Gastroenterol*.

[36] Schmitz F, Tjon JM, Lai Y Identification of a potential physiological precursor of aberrant cells in refractory coeliac disease type II. *Gut*.

[37] Mention JJ, Ahmed MB, Bègue B (2003). Interleukin 15: a key to disrupted intraepithelial lymphocyte homeostasis and lymphomagenesis in celiac disease. *Gastroenterology*.

[38] Colpitts SL, Stoklasek TA, Plumlee CR, Obar JJ, Guo C, Lefrancois L (2012). Cutting edge: the role of IFN-alpha receptor and MyD88 signaling in induction of IL-15 expression in vivo. *The Journal of Immunology*.

[39] Brar P, Lee S, Lewis S, Egbuna I, Bhagat G, Green PHR (2007). Budesonide in the treatment of refractory celiac disease. *American Journal of Gastroenterology*.

[40] Jamma S, Leffler DA, Dennis M (2011). Small intestinal release mesalamine for the treatment of refractory celiac disease type I. *Journal of Clinical Gastroenterology*.

[41] Goerres MS, Meijer JWR, Wahab PJ (2003). Azathioprine and prednisone combination therapy in refractory coeliac disease. *Alimentary Pharmacology & Therapeutics*.

[42] Gillett HR, Arnott IDR, McIntyre M (2002). Successful infliximab treatment for steroid-refractory celiac disease: a case report. *Gastroenterology*.

[43] Costantino G, della Torre A, lo Presti MA, Caruso R, Mazzon E, Fries W (2008). Treatment of life-threatening type I refractory coeliac disease with long-term infliximab. *Digestive and Liver Disease*.

[44] Tack GJ, van Asseldonk DP, van Wanrooij RL, van Bodegraven AA, Mulder CJ (2012). Tioguanine in the treatment of refractory coeliac disease—a single centre experience. *Alimentary Pharmacology & Therapeutics*.

[45] Malamut G, Afchain P, Verkarre V (2009). Presentation and long-term follow-up of refractory celiac disease: comparison of type I with type II. *Gastroenterology*.

[46] Robak T, Wierzbowska A, Robak E (2006). Recent clinical trials of cladribine in hematological malignancies and autoimmune disorders. *Reviews on Recent Clinical Trials*.

[47] Tack GJ, Verbeek WHM, Al-Toma A (2011). Evaluation of cladribine treatment in refractory celiac disease type II. *World Journal of Gastroenterology*.

[48] Al-Toma A, Goerres MS, Meijer JWR (2006). Cladribine therapy in refractory celiac disease with aberrant T cells. *Clinical Gastroenterology and Hepatology*.

[49] Tyndall A, Fassas A, Passweg J (1999). Autologous haematopoietic stem cell transplants for autoimmune disease—feasibility and transplant-related mortality. *Bone Marrow Transplantation*.

[50] Gratwohl A, Passweg J, Bocelli-Tyndall C (2005). Autologous hematopoietic stem cell transplantation for autoimmune diseases. *Bone Marrow Transplantation*.

[51] Passweg J, Tyndall A (2007). Autologous stem cell transplantation in autoimmune diseases. *Seminars in Hematology*.

[52] Al-Toma A, Visser OJ, van Roessel HM (2007). Autologous hematopoietic stem cell transplantation in refractory celiac disease with aberrant T cells. *Blood*.

[53] Tack GJ, Wondergem MJ, Al-Toma A (2011). Auto-SCT in refractory celiac disease type II patients unresponsive to cladribine therapy. *Bone Marrow Transplantation*.

[54] Wahab PJ, Crusius JB, Meijer JW, Uil JJ, Mulder CJ (2000). Cyclosporin in the treatment of adults with refractory coeliac disease—an open pilot study. *Alimentary Pharmacology & Therapeutics*.

[55] Gale J, Simmonds PD, Mead GM, Sweetenham JW, Wright DH (2000). Enteropathy-type intestinal T-cell lymphoma: clinical features and treatment of 31 patients in a single center. *Journal of Clinical Oncology*.

[56] van de Water JMW, Cillessen SAGM, Visser OJ, Verbeek WHM, Meijer CJLM, Mulder CJJ (2010). Enteropathy associated T-cell lymphoma and its precursor lesions. *Best Practice and Research*.

